# Prognostic value of performance status assessed by patients themselves, nurses, and oncologists in advanced non-small cell lung cancer

**DOI:** 10.1054/bjoc.2001.2162

**Published:** 2001-11

**Authors:** M Ando, Y Ando, Y Hasegawa, K Shimokata, H Minami, K Wakai, Y Ohno, S Sakai

**Affiliations:** Department of Preventive Medicine/Biostatistics and Medical Decision Making, First Department of Internal Medicine, Department of Clinical Preventive Medicine, Nagoya University Graduate School of Medicine, Nagoya, 466-8550; National Cancer Center Hospital East, Kashiwa, 277-8577; Department of Internal Medicine, Japanese Red Cross Nagoya First Hospital, Nagoya, 453-8511, Japan

**Keywords:** performance status, oncologist, patient, inter-observer variability, prognostic value

## Abstract

Accuracy in the assessment of performance status by oncologists has not been well evaluated. We investigated possible discrepancies in the assessment of performance status among patients, nurses, and oncologists, and evaluated the prognostic importance of each assessment. Two hundred and six inpatients with inoperable, advanced non-small cell lung cancer were investigated prospectively. Weighted Kappa statistics for inter-observer agreement were 0.53 between oncologists and patients and 0.63 between oncologists and nurses. There was a significant difference among the assessments by the three groups (*P* < 0.001). Oncologists gave the healthiest performance status assessment, nurses an intermediate assessment, and patients the poorest. When included separately in the Cox model, the assessment by each group was significantly correlated with survival. However, the assessment by the patients themselves failed to distinguish survival of patients with performance status 1 and 2. Among the three models including patient-, nurse-, and oncologist-assessed PS, that including oncologist-assessed PS best fitted to the observed survival data. These results showed that the assessment by the patients themselves is different from those by the nurses and the oncologists and provided additional support for the use of the assessment by oncologists in clinical oncology. © 2001 Cancer Research Campaign http://www.bjcancer.com


*British Journal of Cancer* (2001) **85**(11), 1634–1639
© 2001 Cancer Research Campaign
doi: 10.1054/ bjoc.2001.2162, available online at http://www.idealibrary.com on IDEAL

http://www.bjcancer.com

### Prognostic value of performance status assessed by patients themselves, nurses, and oncologists in advanced non-small cell lung cancer

**M Ando[1], Y Ando[2], Y Hasegawa[2], K Shimokata[3], H Minami[4], K Wakai[1], Y Ohno[1] and S Sakai[5]**

[1]Department of Preventive Medicine/Biostatistics and Medical Decision Making; [2]First Department of Internal Medicine; and [3]Department of Clinical Preventive Medicine, Nagoya University Graduate School of Medicine, Nagoya 466-8550; [4]National Cancer Center Hospital East, Kashiwa 277-8577; and [5]Department of Internal Medicine, Japanese Red Cross Nagoya First Hospital, Nagoya 453-8511, Japan

**Summary** Accuracy in the assessment of performance status by oncologists has not been well evaluated. We investigated possible discrepancies in the assessment of performance status among patients, nurses, and oncologists, and evaluated the prognostic importance of each assessment. Two hundred and six inpatients with inoperable, advanced non-small cell lung cancer were investigated prospectively. Weighted Kappa statistics for inter-observer agreement were 0.53 between oncologists and patients and 0.63 between oncologists and nurses. There was a significant difference among the assessments by the three groups ($P$ < 0.001). Oncologists gave the healthiest performance status assessment, nurses an intermediate assessment, and patients the poorest. When included separately in the Cox model, the assessment by each group was significantly correlated with survival. However, the assessment by the patients themselves failed to distinguish survival of patients with performance status 1 and 2. Among the three models including patient-, nurse-, and oncologist-assessed PS, that including oncologist-assessed PS best fitted to the observed survival data. These results showed that the assessment by the patients themselves is different from those by the nurses and the oncologists and provided additional support for the use of the assessment by oncologists in clinical oncology. © 2001 Cancer Research Campaign http://www.bjcancer.com

**Keywords**: performance status; oncologist; patient; inter-observer variability; prognostic value

Performance status (PS) is the assessment of patients' actual level of function and capability of self-care, usually recorded by health professionals using a traditional scoring system such as the Karnofsky scale (Karnofsky et al, 1948) or the Eastern Cooperative Oncology Group (ECOG) scale (Oken et al, 1982). PS has repeatedly been demonstrated to be an important prognostic factor for survival in several major malignancies such as breast cancer (Aisner et al, 1995), ovarian cancer (Lund et al, 1990), small cell lung cancer (Osterlind and Andersen, 1986), and non-small cell lung cancer (Paesmans et al, 1995). Accordingly, it must be taken into consideration in the planning and evaluation of clinical trials of cancer treatment and in the decision on therapeutic options in clinical practice. The subjective view associated with the determination of PS, however, is also well acknowledged (Loprinzi et al, 1994), and there is no 'gold standard' method for the assessment of PS (Schag et al, 1984). Previous studies demonstrated that the inter-observer agreement was only moderate among health professionals such as nurses, social workers, psychiatrists, and oncologists (Hutchinson et al, 1979; Schag et al, 1984; Taylor et al, 1999; Yates et al, 1980).

*Received 15 December 2000*
*Revised 30 July 2001*
*Accepted 17 September 2001*

*Correspondence to:* M Ando, Department of Preventive Medicine/Biostatistics and Medical Decision Making, Nagoya University Graduate School of Medicine, 65 Tsurumai-cho, Showa-ku, Nagoya 466-8550, Japan

Conflicting results have been reported by several studies on inter-observer agreement in grading PS between the patients themselves and the oncologists. A previous study, for example, suggested that the PS assessment by the physicians specialized in clinical oncology was healthier than that by the patients (Loprinzi et al, 1994). However, the result might be biased because the patients had already been assessed by the physicians as chemotherapy-tolerable before participating in simultaneous chemotherapy trials. On the contrary, another small pilot study reported that the patients rated themselves as healtheir than the oncologists did (Taylor et al, 1999). Since patients are supposed to know their own physical condition better than anyone, the assessment of PS by the patients themselves would be reasonable and might be more reliable for prediction and stratification of prognosis. Indeed, recent studies on quality of life have suggested that the information from patient-completed questionnaires could be a prognostic factor independent of PS assessed by the physicians (Coates et al, 1992; Cella et al, 1997). Thus, if there are non-chance discrepancies in the assessments of PS among patients and external observers, the assessment of PS by health professionals should be rationalized by an advantage for prediction and stratification of patients' survival.

We, therefore, planned to investigate whether there was any systematic difference among the assessment of PS by patients themselves, nurses, and oncologists. To elucidate which of the three assessments would be the most useful prognostic factor, we prospectively examined PS of patients with recently diagnosed non-small cell lung cancer and evaluated the prognostic value of each assessment.

### MATERIALS AND METHODS

### Patients

Between January 1995 and November 1998, consecutive inpatients with pathologically confirmed non-small cell lung cancer were prospectively accrued at Japanese Red Cross Nagoya First Hospital, a university-affiliated educational hospital with almost 1000 beds. To be eligible for this study, patients had to have stage III or IV disease at the initial staging procedure described below. Of 209 patients who were eligible, 3 refused to enter the study because of no interest in participation. This study had at least 85% statistical power to detect the 15% difference in the proportions of patients assessed as ECOG PS 0 to 1 (estimated proportions, 55% for patients themselves, 70% for oncologists) at the 5% significance level. This study was approved by the Institutional Review Board, and participants gave written informed consent before they entered the study.

### Performance status assessment

The patients were asked to complete a short questionnaire regarding their own assessment of ECOG PS (Oken et al, 1982) as soon as after the diagnosis of lung cancer (Table 1). The questionnaire was delivered to each patient by a ward clerk to be filled out by the patient at the bedside. When the patient was unable to fill out the questionnaire by himself/herself, the clerk read it aloud and wrote down the patient's answer given orally. The clerk did not provide any specific implication of the terms in the questionnaire that might cause bias in the final interpretation of each PS description. Simultaneously, the attending oncologist and nurse working on the ward assessed the patient's ECOG PS in separate rooms of the ward by completing each questionnaire which included the same description of PS as shown in Table 1. The completed questionnaires were sealed in envelopes by each respondent before being turned in to the responsible investigator of this study (MA). Thus, each observer was blinded to the ratings of the others. The nurses and oncologists, who were dedicated to the care of patients with lung cancer, received no special training for participation in this study because they were familiar with the assessment of PS in daily oncology practice. They were asked not to interview the patients solely for assessment of PS, but to use the information they had obtained during the patient's care. All the oncologists were members of the Japan Lung Cancer Society.

### Staging procedures

Patients were staged according to the TNM system (Mountain, 1997). Patients underwent the following procedures on the initial presentation: medical history, physical examination, histologic or cytologic confirmation of non-small cell lung cancer, complete blood cell counts with differential, serum chemistry, chest radiograph and computed tomography (CT), abdominal CT and/or ultrasonography, brain CT, and radionuclide bone scan. Bone radiographs were used to confirm metastatic lesions suspected by bone scan.

### Demographic and clinical factors

Besides PS assessments by patients, nurses, and oncologists, the following potential prognostic factors were evaluated: age, gender, histology, metastatic organs (brain, liver, bone, lung, and lymph node), disease stage, T factor, N factor, weight loss within 6 months, leukocyte count, neutrophil count, platelet count, haemoglobin, serum albumin, serum lactate dehydrogenase (LDH), and the treatment modality (chemoradiotherapy, chemotherapy, radiotherapy, or supportive care alone).

### Statistical methods

The proportions of patients assessed as fully ambulatory (PS 0 or 1) by the patients themselves and the oncologists were compared by chi-square test. The inter-observer agreement in the assessment of PS was evaluated using simple and weighted Kappa statistics (Cohen, 1960, 1968). In order to identify the predictor of disagreement in the PS assessment between the patients themselves and the oncologists, a multivariate logistic regression analysis was performed (Breslow and Day, 1980). In this analysis, the following variables were investigated for association with the proportion of patients whose assessment agreed with that of the oncologists: age, gender, weight loss, disease stage, treatment modality, and attending oncologist. Friedman test and Mann-Whitney U-test were used to compare the assessments of PS by the three groups and to compare each of the three pairs of assessments, respectively. Because elderly patients are generally excluded from clinical trials of cancer treatment, we examined whether the results of these comparisons remain unchanged when patients over 75 years old were excluded from the study population. Survival was calculated from the day of pathological diagnosis until death. Patients alive were censored as of last known follow-up. Survival curves were calculated using Kaplan-Meier method and compared using log-rank test. Prognostic importance of the factors including PS assessed by the three groups was analyzed using the Cox regression model (Cox, 1972). Forward stepwise procedures were used to select factors that were included in the final model. A-2 log likelihood value for fitting a model with all the explanatory variables was

**Table 1** Patient questionnaire for the determination of their ECOG PS

| | |
|---|---|
| Please circle the number of the phrase which characterizes you best at this time, according to your own judgement. Please do not ask advice from any others | |
| 0 | Fully active, able to carry on all pre-disease performance without restriction |
| 1 | Restricted in physically strenuous activity but ambulatory and able to carry out work of a light or sedentary nature, e.g., light house work, office work |
| 2 | Ambulatory and capable of all self-care but unable to carry out any work activities. Up and about more than 50% of waking hours |
| 3 | Capable of only limited self-care, confined to bed or chair more than 50% of waking hours |
| 4 | Completely disabled. Cannot carry on any self-care. Totally confined to bed or chair |

ECOG, Eastern Cooperative Oncology Group; PS, performance status.

*© 2001 Cancer Research Campaign*

calculated for individual Cox models which included patient-, nurse-, and oncologist-assessed PS separately, and was used to compare the performance of these three models. The likelihood ratio test was used to examine whether the fit of model including oncologist-assessed PS, which proved to be the best performing model in the present study, could be further improved by additional inclusion of the PS scores by the patients or the nurses. Statistical analyses were performed by SAS ver. 6.12 software (SAS Institute Inc., Cary, NC).

### RESULTS

Characteristics of the 206 patients who entered the study were presented in Table 2. Age of the subjects was 67 ± 10 (mean ± SD) years old. No patient was lost to follow-up, and 16 patients alive were censored. The median follow-up time in the 16 patients was 824 days (range 352–1296).

Twelve oncologists and 45 nurses participated in the assessment of PS. The distribution of the assessments by the patients themselves and the health professionals are shown in Tables 3 and 4. The oncologists assessed 71% of patients as ECOG PS 0 or 1, while 59% of the patients rated themselves as ECOG PS 0 or 1 ($P = 0.007$). The simple and weighted Kappa statistics between the two assessment were 0.29 (95% confidence interval 0.20–0.39) and 0.53 (95% confidence interval 0.45–0.61). The two Kappa values were 0.45 (95% confidence interval 0.36–0.55) and 0.63 (95% confidence interval 0.54–0.71) between the nurses and the oncologists, and 0.38 (95% confidence interval 0.28–0.47) and 0.56 (95% confidence interval 0.48–0.64) between the patients and the nurses, respectively. Thus, the best agreement was observed between nurse- and oncologist-assessed PS irrespective of the Kappa statistics used. In multivariate logistic regression analysis, only gender was significantly correlated with the disagreement in the PS assessment between the patients themselves and the oncologists ($P = 0.037$); the level of agreement was lower in female patients (37.2%) than in male patients (54.0%). The proportion of patients whose assessment of PS was poorer than that by the oncologists was 51.2% in females and 31.3% in males ($P = 0.015$).

A systematic difference in the assessments of PS was observed among the three groups ($P < 0.001$); assessment by oncologists

**Table 3** Agreement in assessment of ECOG PS between patients and oncologists ($n = 206$)

| Oncologist's score | Patient's score 0 | 1 | 2 | 3 | 4 |
|---|---|---|---|---|---|
| 0 | 17 | 22 | 0 | 0 | 0 |
| 1 | 13 | 63 | 23 | 8 | 1 |
| 2 | 1 | 3 | 6 | 12 | 2 |
| 3 | 0 | 1 | 4 | 11 | 5 |
| 4 | 0 | 1 | 0 | 6 | 7 |

ECOG, Eastern Cooperative Oncology Group; PS, performance status.

**Table 4** Agreement in assessment of ECOG PS between patients and nurses ($n = 206$)

| Nurse's score | Patient's score 0 | 1 | 2 | 3 | 4 |
|---|---|---|---|---|---|
| 0 | 15 | 12 | 0 | 0 | 0 |
| 1 | 14 | 71 | 19 | 10 | 2 |
| 2 | 2 | 5 | 8 | 8 | 0 |
| 3 | 0 | 2 | 6 | 17 | 6 |
| 4 | 0 | 0 | 0 | 2 | 7 |

ECOG, Eastern Cooperative Oncology Group; PS, performance status.

**Table 2** Demographic and clinical variables of patients

| | Number of patients | | | | | | | Median survival[a] (days) |
|---|---|---|---|---|---|---|---|---|
| | Total | Patient-assessed PS (%) | | Nurse-assessed PS (%) | | Oncologist-assessed PS (%) | | |
| | | 0–1 | 2–4 | 0–1 | 2–4 | 0–1 | 2–4 | |
| Gender | | | | | | | | |
| female | 43 | 21 (49) | 22 (51) | 27 (63) | 16 (37) | 29 (67) | 14 (33) | 245 |
| male | 163 | 100 (61) | 63 (39) | 116 (71) | 47 (29) | 118 (72) | 45 (28) | 210 |
| Weight loss within 6 months | | | | | | | | |
| < 5% | 115 | 83 (72) | 32 (28) | 95 (83) | 20 (17) | 100 (87) | 15 (13) | 280 |
| 5–10% | 46 | 22 (48) | 24 (52) | 27 (59) | 19 (41) | 26 (57) | 20 (43) | 181 |
| ≥ 10% | 45 | 16 (36) | 29 (64) | 21 (47) | 24 (53) | 21 (47) | 24 (53) | 90 |
| Disease stage | | | | | | | | |
| IIIA | 34 | 26 (76) | 8 (24) | 31 (91) | 3 (9) | 30 (88) | 4 (12) | 463 |
| IIIB | 85 | 53 (62) | 32 (38) | 58 (68) | 27 (32) | 62 (73) | 23 (27) | 255 |
| IV | 87 | 42 (48) | 45 (52) | 54 (62) | 33 (38) | 55 (63) | 32 (37) | 106 |
| Histology | | | | | | | | |
| squamous cell carcinoma | 84 | 55 (65) | 29 (35) | 63 (75) | 21 (25) | 63 (75) | 21 (25) | 244 |
| adenocarcinoma | 108 | 61 (56) | 47 (44) | 72 (56) | 36 (44) | 77 (71) | 31 (29) | 216 |
| large cell carcinoma | 14 | 5 (36) | 9 (64) | 8 (57) | 6 (43) | 7 (50) | 7 (50) | 66 |
| Treatment | | | | | | | | |
| chemoradiotherapy | 31 | 28 (90) | 3 (10) | 30 (97) | 1 (3) | 30 (97) | 1 (3) | 476 |
| chemotherapy | 55 | 41 (75) | 14 (25) | 47 (85) | 8 (15) | 51 (93) | 4 (7) | 255 |
| radiotherapy | 20 | 11 (55) | 9 (45) | 13 (65) | 7 (35) | 13 (65) | 7 (35) | 198 |
| supportive care | 100 | 41 (41) | 59 (59) | 53 (53) | 47 (47) | 53 (53) | 47 (47) | 96 |

[a]Median survival was calculated after exclusion of four patients with previous malignancy.
PS, performance status.

 *© 2001 Cancer Research Campaign*

was the healthiest, that by nurses was intermediate, and that by patients themselves was the poorest. Assessments by oncologists and nurses were both significantly healthier than that by patients themselves ($P < 0.001$ and $P = 0.003$, respectively). On the other hand, there was no significant difference in the assessment between nurses and oncologists ($P = 0.137$). Difference among the three assessments remained significant when female and male patients were analysed separately ($P < 0.001$ and $P = 0.004$, respectively). The systematic difference remained significant when 23 patients over 75 years old were excluded ($P < 0.001$), with healthier assessments by oncologists and nurses than those by patients ($P < 0.001$ and $P = 0.005$, respectively).

In survival analysis, 4 patients with previous malignancy were excluded. Median survival time of the resultant 202 patients was 221 days. Survival curves according to PS graded by patients and oncologists are shown in Figure 1. In univariate analyses, PS assessments by patients, nurses, and oncologists were all significantly correlated with survival ($P < 0.001$ in all cases). However, survival curves of patients with PS 1 and 2 by their own assessment were superimposed until 600 days after diagnosis. When included separately in the Cox model, each of the three PS assessments was significantly correlated with survival (Table 5). Gender, weight loss, haemoglobin, disease stage, T factor, bone metastasis, and treatment modality were selected as covariates in every case. In addition, lymph node metastasis was selected as a covariate when nurse- or oncologist-assessed PS was incorporated into the model. Because the selected factors were almost the same across the three models, we used all these factors as covariates for which the adjustment was carried out. The –2 log likelihood values with all the explanatory variables were 1494.4, 1502.2, and 1488.2 for the Cox models including patient-, nurse-, and oncologist-assessed PS, respectively. Since the degrees of freedom were the same among the three models, these results indicated that the Cox model including oncologist-assessed PS best fitted to the observed survival data.

**Table 5** Prognostic value of ECOG PS graded by patients, nurses, and oncologists: results of analyses separately included in the Cox model

| | Multivariate-adjusted hazard ratios[a] (95% CI) | | |
|---|---|---|---|
| **Variables** | **Patient-assessed PS** | **Nurse-assessed PS** | **Oncologist-assessed PS** |
| PS Score | | | |
| 0 | reference | reference | reference |
| 1 | 1.63 (0.97–2.83) | 1.79 (1.07–3.12) | 1.63 (1.04–2.64) |
| 2 | 1.60 (0.89–2.93) | 2.08 (1.06–4.13) | 1.95 (1.00–3.79) |
| 3 | 2.77 (1.46–5.33) | 2.25 (1.15–4.49) | 4.28 (2.23–8.10) |
| 4 | 3.90 (1.81–8.25) | 2.99 (1.18–7.16) | 3.43 (1.61–7.07) |
| *P*-trend | < 0.001 | 0.018 | < 0.001 |

[a]In each model, adjustment was made for gender, weight loss, haemoglobin, T factor, lymph node metastasis, bone metastasis, disease stage, and treatment modality.
ECOG, Eastern Cooperative Oncology Group; PS, performance status; CI, confidence interval.

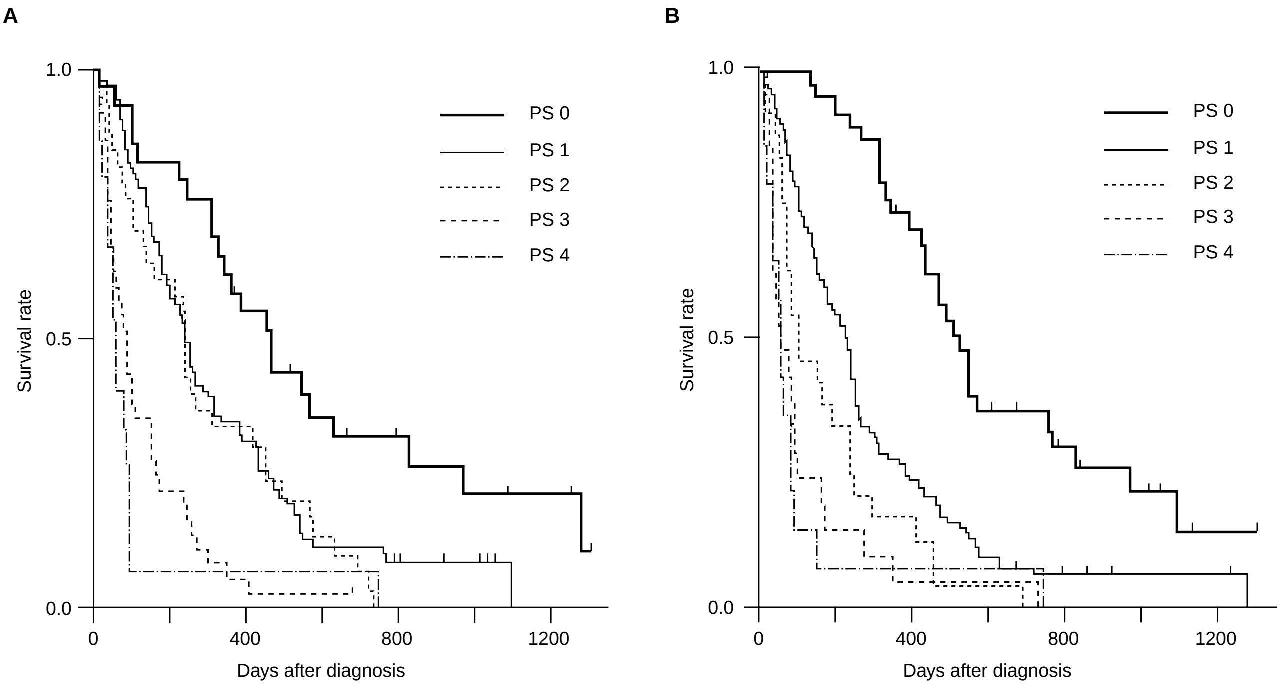

**Figure 1** Kaplan-Meier plots of survival of patients with advanced non-small cell lung cancer, divided by the assessment of performance status (**A**) by the patients themselves and (**B**) by the oncologists

*© 2001 Cancer Research Campaign*

The fit of this oncologist-assessed PS model was not significantly improved by additional inclusion of categorical variables indicating the PS scores by the patients or the nurses (*P* = 0.374 and *P* = 0.339, respectively).

### DISCUSSION

The present study demonstrated that the assessments of PS by health professionals, i.e., nurses and oncologists, were systematically different from that by the patients themselves. This finding may be relevant to previous reports that observer ratings of patients' quality of life were different from those of the patients themselves (Presant, 1984; Slevin et al, 1988; Osoba, 1994). Assessments of PS by nurses and oncologists were found to be healthier than that by the patients themselves. Furthermore, the agreement in the PS assessment between the two health professionals was better than that between the patients and the health professionals. These results may suggest that the tendency to rate healthier PS was not peculiar to the oncologists but also true of other health professionals. The difference in the PS assessment remained significant when the analyses were limited to younger patients who are considered potentially suitable for clinical trials. We, therefore, consider that the problem of discrepancy in the assessment of PS would be relevant in judging eligibility for clinical trials.

Investigations of the predictor variables of inter-observer disagreement are scarce in the PS assessment, although a similar analysis was conducted in the assessment of quality of life in cancer patients (Sneeuw et al, 1999). Interestingly, the results of the present study showed that female patients had a lower level of agreement in the PS assessment between the patients and the oncologists. The female patients reported poorer PS of their own compared with the male patients. Only two female oncologists assessed PS of 22 patients in our study, and this small number precluded detailed analysis of the gender-related difference. However, a similar difference was reported in other studies on quality of life in the general population (Brazier et al, 1992; Jenkinson et al, 1993; Hjermstad et al, 1998). In these studies, females reported poorer health status, including physical well-being, than males. In addition to the results in these 'reference population', female patients with asthma were shown to report worse physical and social status than male patients (Osborne et al, 1998), which warrants further studies to investigate whether this gender-related difference is also observed in patients with other chronic diseases.

Oncologist-assessed PS has traditionally been used as a criterion in clinical trials, which is partly supported by an acceptable level of agreement among the assessments by oncologists, especially for patients in good physical condition (Conill et al, 1990; Roila et al, 1991; Sørensen et al, 1993). This is the first study investigating whether the assessment of PS by the oncologists is rationalized in terms of the prognostic value. Univariate and multivariate analyses demonstrated that the assessment of PS 0, 1, and 2 by oncologists successfully stratified survival, whereas that by the patients themselves failed to distinguish survival of patients with PS 1 and 2. It would be critical to stratify patients for clinical trials in which only fully ambulatory patients should be the candidates (Bonomi et al, 1991; Ihde, 1992; Shepherd, 1994). Furthermore, the Cox model including oncologist-assessed PS best fitted to the observed survival data as indicated by the lowest -2 log likelihood value. These advantages for prediction and stratification of prognosis provided additional support for the use of the assessment of PS by oncologists in clinical trials for advanced non-small cell lung cancer. Because one reason for restriction on the entry of patients with PS 2 onto clinical trials were their poor tolerability of treatment (Ruckdeschel et al, 1986), further work will be needed to determine whether oncologist-assessed PS is also more reliable in predicting treatment-related toxicities than patient-assessed PS.

The present study showed that the straightforward assessment of PS by the patients themselves is not a fruitful method, because the assessment by the patients failed to demonstrate a better prognostic value than that by the oncologists. Although Loprinzi et al suggested that patient-assessed PS could provide independent prognostic information in their study, the ability of the Cox model to explain the data did not change substantially even when PS assessed by patients themselves was included in or excluded from the model (Loprinzi et al, 1994). In addition, the poorest model in their study was the one that did not include oncologist-assessed PS. These results suggest that the prognostic importance of PS assessment by patients themselves does not exceed that by oncologists. This advantage in assessment by oncologists in the estimation of prognosis might be partly explained by the availability of information from observation and examination of the patient (Schag et al, 1984). Considering the problem of colinearity among the PS assessments, prognostic importance of the combination indices of these assessments may be worthy of exploration.

In conclusion, the assessment of PS by the patients themselves is different from the assessments by health professionals such as nurses and oncologists. Assessments by the oncologists and the nurses were significantly healthier than that by patients themselves. However, this does not imply that health professionals would be optimistic and patients pessimistic and it is not possible to say who is correct. This might be an unconscious appeal for comfort or care from patients toward health professionals. Rather, this suggests that the assessments by health professionals would predict patients' prognosis more precisely than that by the patients themselves. Our results reinforced the rationale for the traditional use of oncologist-assessed PS in clinical oncology, and supported treatment decisions based on oncologists' assessment of PS.

### ACKNOWLEDGEMENT

This work was supported in part, by a Grant-in-Aid for Cancer Research (7–30) from the Ministry of Health and Welfare of Japan.

### REFERENCES

Aisner J, Cirrincione C, Perloff M, Perry M, Budman D, Abrams J, Panasci L, Muss H, Citron M and Holland J (1995) Combination chemotherapy for metastatic or recurrent carcinoma of the breast–a randomized phase III trial comparing CAF versus VATH versus VATH alternating with CMFVP: Cancer and Leukemia Group B Study 8281. *J Clin Oncol* **13**: 1443–1452

Bonomi P, Gale M, Rowland K, Taylor IV SG, Purl S, Reddy S, Lee MS, Phillips A, Kittle CF, Warren W and Faber LP (1991) Pre-treatment prognostic factors in stage III non-small cell lung cancer patients receiving combined modality treatment. *Int J Radiat Oncol Biol Phys* **20**: 247–252

Brazier JE, Harper R, Jones NM, O'Cathain A, Thomas KJ, Usherwood T and Westlake L (1992) Validating the SF-36 health survey questionnaire: new outcome measure for primary care. *BMJ* **305**: 160–164

Breslow NE and Day NE (1980) Unconditional logistic regression for large strata. In: *The Analysis of Case-control Studies, Statistical Methods in Cancer Research, vol. 1,* Davis W (ed.) pp 192–247. International Agency for Research on Cancer: Lyon

 *© 2001 Cancer Research Campaign*

Cella D, Fairclough DL, Bonomi PB, Kim K and Johnson D (1997) Quality of life in advanced non-small cell lung cancer: results from eastern cooperative oncology group study E5592. *Proc Am Soc Clin Oncol* **16**: 2a

Coates A, Gebski V, Signorini D, Murray P, McNeil D, Byrne M and Forbes JF (1992) Prognostic value of quality-of-life scores during chemotherapy for advanced breast cancer. Australian New Zealand Breast Cancer Trials Group. *J Clin Oncol* **10**: 1833–1838

Cohen J (1960) A coefficient of agreement for nominal scales. *Educ Psychol Meas* **20**: 37–46

Cohen J (1968) Weighted kappa: nominal scale agreement with provision for scale disagreement or partial credit. *Psychol Bull* **70**: 213–220

Conill C, Verger E and Salamero M (1990) Performance status assessment in cancer patients. *Cancer* **65**: 1864–1866

Cox DR (1972) Regression models and life-tables (with discussions). *J R Stat Soc B* **34**: 187–220

Hjermstad MJ, Fayers PM, Bjordal K and Kaasa S (1998) Health-related quality of life in the general Norwegian population assessed by the European Organization for Research and Treatment of Cancer Core Quality-of-Life Questionnaire: the QLQ = C30 (+ 3). *J Clin Oncol* **16**: 1188–1196

Hutchinson TA, Boyd NF, Feinstein AR, Gonda A, Hollomby D and Rowat B (1979) Scientific problems in clinical scales, as demonstrated in the Karnofsky index of performance status. *J Chronic Dis* **32**: 661–666

Ihde DC (1992) Chemotherapy of lung cancer. *N Engl J Med* **327**: 1434–1441

Jenkinson C, Coulter A and Wright L (1993) Short form 36 (SF36) health survey questionnaire: normative data for adults of working age. *BMJ* **306**: 1437–1440

Karnofsky DA, Ableman WH, Craver LF and Burchenal JH (1948) The use of nitrogen mustard in the palliative treatment of carcinoma. *Cancer* **1**: 634–656

Loprinzi CL, Laurie JA, Wieand HS, Krook JE, Novotny PJ, Kugler JW, Bartel J, Law M, Bateman M, Klatt NE, Dose AM, Etzell PS, Nelimark RA, Mailliard JA and Moertel CG for the North Central Cancer Treatment Group (1994) Prospective evaluation of prognostic variables from patient-completed questionnaires. *J Clin Oncol* **12**: 601–607

Lund B, Williamson P, van Houwelingen HC and Neijt JP (1990) Comparison of the predictive power of different prognostic indices for overall survival in patients with advanced ovarian carcinoma. *Cancer Res* **50**: 4626–4629

Mountain CF (1997) Revisions in the International System for Staging Lung Cancer. *Chest* **111**: 1710–1717

Oken MM, Creech RH, Tormey DC, Horton J, Davis TE, McFadden ET and Carbone PP (1982) Toxicity and response criteria of the Eastern Cooperative Oncology Group. *Am J Clin Oncol* **5**: 649–655

Osborne ML, Vollmer WM, Linton KL and Buist AS (1998) Characteristics of patients with asthma within a large HMO: a comparison by age and gender. *Am J Respir Crit Care Med* **157**: 123–128

Osoba D (1994) Lessons learned from measuring health-related quality of life in oncology. *J Clin Oncol* **12**: 608–616

Osterlind K and Andersen PK (1986) Prognostic factors in small cell lung cancer: multivariate model based on 778 patients treated with chemotherapy with or without irradiation. *Cancer Res* **46**: 4189–4194

Paesmans M, Sculier JP, Libert P, Bureau G, Dabouis G, Thiriaux J, Michel J, Van Cutsem O, Sergysels R, Mommen P and Klastersky J for the European Lung Cancer Working Party (1995) Prognostic factors for survival in advanced non-small-cell lung cancer: univariate and multivariate analyses including recursive partitioning and amalgamation algorithms in 1,052 patients. *J Clin Oncol* **13**: 1221–1230

Presant CA (1984) Quality of life in cancer patients. Who measures what? *Am J Clin Oncol* **7**: 571–573

Roila F, Lupattelli M, Sassi M, Basurto C, Bracarda S, Picciafuoco M, Boschetti E, Milella G, Ballatori E, Tonato M and Favero AD (1991) Intra and interobserver variability in cancer patients' performance status assessed according to Karnofsky and ECOG scales. *Ann Oncol* **2**: 437–439

Ruckdeschel JC, Finkelstein DM, Ettinger DS, Creech RH, Mason BA, Joss RA and Vogl S (1986) A randomized trial of the four most active regimens for metastatic non-small-cell lung cancer. *J Clin Oncol* **4**: 14–22

Schag CC, Heinrich RL and Ganz PA (1984) Karnofsky performance status revisited: reliability, validity, and guidelines. *J Clin Oncol* **2**: 187–193

Shepherd FA (1994) Treatment of advanced non-small cell lung cancer. *Semin Oncol* **21**: S7-7–S7-18

Slevin ML, Plant H, Lynch D, Drinkwater J and Gregory WM (1988) Who should measure quality of life, the doctor or the patient? *Br J Cancer* **57**: 109–112

Sneeuw KC, Aaronson NK, Sprangers MA, Detmar SB, Wever LD and Schornagel JH (1999) Evaluating the quality of life of cancer patients: assessments by patients, significant others, physicians and nurses. *Br J Cancer* **81**: 87–94

Sørensen JB, Klee M, Palshof T and Hansen HH (1993) Performance status assessment in cancer patients. An inter-observer variability study. *Br J Cancer* **67**: 773–775

Taylor AE, Olver IN, Sivanthan T, Chi M and Purnell C (1999) Observer error in grading performance status in cancer patients. *Support Care Cancer* **7**: 332–335

Yates JW, Chalmer B and McKegney FP (1980) Evaluation of patients with advanced cancer using the Karnofsky performance status. *Cancer* **45**: 2220–2224

*© 2001 Cancer Research Campaign*
